# Comparative analysis of the susceptibility of *Aedes aegypti* and Japanese *Aedes albopictus* to all dengue virus serotypes

**DOI:** 10.1186/s41182-023-00553-5

**Published:** 2023-11-02

**Authors:** Daisuke Kobayashi, Izumi Kai, Astri Nur Faizah, Meng Ling Moi, Shigeru Tajima, Tomohiko Takasaki, Toshinori Sasaki, Haruhiko Isawa

**Affiliations:** 1 Department of Medical Entomology, National Institute of Infectious Diseases, Tokyo, Japan; 2 Management Department of Biosafety, Laboratory Animal, and Pathogen Bank, National Institute of Infectious Diseases, Tokyo, Japan; 3 Graduate School of Agriculture, Meiji University, Kanagawa, Japan; 4 Department of Virology I, National Institute of Infectious Diseases, Tokyo, Japan; 5 Present Address: Department of Developmental Medical Sciences, Graduate School of Medicine, The University of Tokyo, Tokyo, Japan; 6 Present Address: BML, Inc., Tokyo, Japan

**Keywords:** Dengue, DENV, DENV-1, Mosquito, *Aedes*, *Aedes albopictus*, Asian tiger mosquito, *Aedes aegypti*, Susceptibility, Japan

## Abstract

**Background:**

Dengue fever, caused by the dengue virus (DENV), is the most common viral infection transmitted by *Aedes* mosquitoes (mainly *Ae. aegypti* and *Ae. albopictus*) worldwide. *Aedes aegypti* is not currently established in Japan, and *Ae. albopictus* is the primary vector mosquito for DENV in the country, but knowledge of its viral susceptibility is limited. Therefore, we aimed to clarify the status of DENV susceptibility by comparing the infection and dissemination dynamics of Japanese *Ae. albopictus* to all known DENV serotypes with those of *Ae. aegypti*.

**Methods:**

After propagation of each DENV serotype in Vero cells, the culture supernatants were mixed with defibrinated rabbit blood and adenosine triphosphate, and the mixture was artificially blood-sucked by two colonies of *Ae. albopictus* from Japan and one colony of *Ae. aegypti* from a dengue-endemic country (Vietnam). After 14 days of sucking, the mosquito body was divided into two parts (thorax/abdomen and head/wings/legs) and total RNA was extracted from each sample. DENV RNA was detected in these extracted RNA samples using a quantitative RT-PCR method specific for each DENV serotype, and infection and dissemination rates were analyzed.

**Results:**

The Japanese *Ae. albopictus* colonies were susceptible to all DENV serotypes. Its infection and dissemination rates were significantly lower than those of *Ae. aegypti*. However, the number of DENV RNA copies in *Ae. albopictus* was almost not significantly different from that in *Ae. aegypti*. Furthermore, Japanese *Ae. albopictus* differed widely in their susceptibility to each DENV serotype.

**Conclusions:**

In Japanese *Ae. albopictus*, once DENV overcame the midgut infection barrier, the efficiency of subsequent propagation and dissemination of the virus in the mosquito body was comparable to that of *Ae. aegypti*. Based on the results of this study and previous dengue outbreak trends, *Ae. albopictus* is predicted to be highly compatible with DENV-1, suggesting that this serotype poses a high risk for future epidemics in Japan.

**Supplementary Information:**

The online version contains supplementary material available at 10.1186/s41182-023-00553-5.

## Background

Dengue fever, caused by infection with dengue virus (DENV), is mainly endemic to tropical and subtropical regions of the world and is the most common mosquito-borne viral infection [1]. There are four serotypes of DENV (DENV-1, DENV-2, DENV-3, and DENV-4). A heteroserotype DENV secondary infection (different serotype from the primary infection) is the greatest risk factor for severe dengue, which can lead to organ failure and death [2]. DENV is maintained in nature through transmission between mosquitoes and vertebrates, including humans. In urban area, DENV is transmitted between urban *Aedes* mosquitoes (*Aedes aegypti* and *Ae. albopictus*) and humans [3]. Of the two vector species involved in the urban cycle of DENV transmission, the *Ae. aegypti* mosquito is considered the primary vector [4]. This is thought to be due to the higher vectorial capacity of DENV and the unique ecology of the species (high blood-sucking preference for humans, living in human dwellings, etc.), which increases the efficiency of DENV transmission [4]. In contrast, *Ae. albopictus* prefers vegetated environments and typically suck blood from various animals, including humans [5–7]. The distribution of *Ae. albopictus* is wider than that of *Ae. aegypti*, ranging from tropical to temperate regions. Therefore, in temperate regions where *Ae. aegypti* is absent, *Ae. albopictus* is the main vector of DENV. Outbreaks in which this species was the sole vector have recently been reported in several temperate regions worldwide (Table 1). Even in tropical and subtropical regions, there are areas where *Ae. albopictus* is the dominant species, and relatively large dengue epidemics have also been reported from the US state of Hawaii and China [8–10]. Thus, these cases demonstrate the potential of *Ae. albopictus* to spread DENV at the same level as the main vector mosquito, *Ae. aegypti*.

**Table 1:** Autochthonous transmission of dengue virus by *Aedes albopictus* mosquitoes in the temperate zone from 2010 to 2022

Country	Year	Location of autochthonous transmission	Number of cases	Serotype	References
Croatia	2010	Korčula Island and the Pelješac peninsula	10	1	[40, 41]
France	2010	Alpes-Maritimes department	2	1	[42]
	2013	Bouches-du-Rhône department	1	2	[43]
	2014	Bouches-du-Rhône and Var departments	4	1 and 2	[44]
	2015	Gard department	7	1	[45]
	2018	Alpes-Maritimes, Gard, and Hérault departments	8	1 and 2	[44]
	2019	Alpes-Maritimes and Rhône departments	9	1	[46]
	2020	Alpes-Maritime, Gard, Hérault, and Var departments	13	NA*	[46]
	2021	Hérault and Var departments	2	NA	[46]
	2022	Alpes-Maritime, Corsica, Haute-Garonne, Hautes-Pyrénées, Pyrénées-Orientales, Tarn et Garonne, and Var departments	65	1 and 3	[47]
Italy	2020	Veneto region	11	1	[48]
Japan	2014	Tokyo	162	1	[15]
	2019	Kyoto or Nara**	3	2	[16]
Spain	2018	Catalonia region, Murcia region or province of Cadiz	6	1	[49, 50]
	2019	Catalonia region	1	NA	[46]
	2022	Ibiza	6	NA	[46]

^*^information was not available

**presumed infection site

Much of Japan’s land area lies in a temperate zone, and *Ae. aegypti* is not currently established in the country [11]. Several autochthonous outbreaks of dengue have been reported in Japan, but imported cases typically initiate every epidemic as the virus is not native to the country [12]. The most recent large dengue outbreak in Japan occurred in Tokyo in 2014 [13]. During this outbreak, the virus was transmitted by *Ae. albopictus* [13–15]. This outbreak ultimately resulted in 162 reported cases [15], the highest number of cases reported in recent dengue outbreaks in temperate regions (Table 1). Additionally, cases of other autochthonous dengue infections were reported also in 2019 [16]. Furthermore, approximately over 70 years prior to these outbreaks, during World War II, Japan experienced a large-scale domestic dengue epidemic, and *Ae. albopictus* was the main vector at that time (reviewed by Kurihara [17]).

Several fragmentary studies have investigated the susceptibility and vectorial capacity of Japanese *Ae. albopictus* to DENVs [18–22]. However, no study has compared the susceptibility of Japanese *Ae. albopictus* to all DENV serotypes using the same mosquito strain or colony, nor compared it with that of *Ae. aegypti*. Therefore, we aimed to clarify the status of DENV susceptibility in a unified manner by comparing the infection and dissemination dynamics of Japanese *Ae. albopictus* to all DENV serotypes with those of *Ae. aegypti*.

## Methods

### Mosquito colonies

Two colonies of Japanese *Ae. albopictus* were used in this study. The colony named IKT was derived from individuals collected in Kawasaki City, Kanagawa Prefecture (Japan) in 2008 (Table 2) [20]. The colony was subsequently reared in the laboratory for more than 50 generations since field collection. This colony was found to be susceptible to DENV-1 and DENV-2 in a previous study [20]. The other *Ae. albopictus* colony used in this study was individuals of the third generation since collection in Numata City, Gumma Prefecture, Japan (colony name BSD; Table 2). Because of the possibility that foreign *Ae. albopictus* populations are collected near ports and international airports [23, 24], we used *Ae. albopictus* collected in Numata City (inland and without nearby airports). In addition, *Ae. aegypti* mosquitoes collected in Ho Chi Minh City, Vietnam, a dengue-endemic area, were used as positive controls (designated HCM; Table 2) [25]. These mosquito colonies were fed a ground diet (Oriental Yeast Industry, Tokyo, Japan) during the larval stage, and adults reared on a 3% sucrose solution. Females were fed mouse blood and allowed to lay eggs. Both larvae and adults were reared at 25 °C and 70% humidity with a 16 h light (L):8 h dark (D) cycle.

**Table 2:** Mosquito colonies used in this study

Species	Colony name	Collection site	Year of collection	Generation
*Aedes aegypti*	HCM	Ho Chi Minh City, Viet Nam	2016	28th
*Aedes albopictus*	IKT	Kawasaki City, Kanagawa Prefecture, Japan	2008	Unknown (more than 50th)
	BSD	Numata City, Gunma Prefecture, Japan	2022	3rd

### Dengue viruses

DENV strains obtained during the autochthonous outbreak [26] or derived from imported cases [27, 28] were used in all experiments (Table 3). Each virus was propagated in Vero cells (derived from African green monkeys; Department of Veterinary Science, National Institute of Infectious Diseases, Japan) before the experiment. Viral titers were determined by a focus-forming assay using the same method as described in a previous study [25].

**Table 3:** Dengue viruses used in this study

Serotype	Genotype	Strain	Isolation source	Country of isolated	Year of isolation	Accession no	Virus titer*
1	I	D1/Hu/Saitama/NIID100/2014	Human patient	Japan	2014	LC011945	7.17 × 10^6^ FFU**/mL
2	Cosmopolitan (Indian Sub-continent lineage)	D2/Hu/India/NIID74/2009	Human patient	Japan (imported case from India)	2009	LC367234	1.08 × 10^6^ FFU/mL
3	II	D3/Hu/Thailand/NIID040/2000	Human patient	Japan (imported case from Thailand)	2000	AB111082	0.72 × 10^6^ FFU/mL
4	II	D4/Hu/Marshall Islands/NIID30/2012	Human patient	Japan (imported case from Marshall Island)	2012	AB710464	3.20 × 10^6^ FFU/mL

^*^titers in bloodmeal used for infection experiment

^**^focus forming units

### Infection experiment

Infection experiments were performed similarly to those described in our previous studies [25, 29]. Briefly, a mixture of the culture supernatant containing DENV, rabbit defibrinated blood (Nippon Biotest Laboratories. Inc., Tokyo, Japan), and adenosine triphosphate (final concentration, 3 mM) (Fujifilm Wako Pure Chemical, Osaka, Japan) was prepared, and artificial blood-sucking performed using the Hemotek 5W1 membrane feeding system for blood-sucking insects (Hemotek Ltd., Blackburn, UK). Adult females within 10 days after emergence that had fasted overnight were allowed to feed on blood for 1 h. Only fully fed individuals were sorted under a stereomicroscope and used in subsequent experiments. Engorged mosquitoes were kept in a cage containing a 3% sucrose solution at 28 °C with a 16L:8D cycle. To facilitate normal physiology and metabolism, an oviposition tray was placed in the cage on which the mosquitoes were allowed to lay eggs. Under these conditions, the mosquitoes were maintained for 14 days after blood-feeding.

### Quantitative measurement of dengue virus RNA in mosquito body parts

To measure the dynamics of DENV propagation in mosquitoes, the copy number of DENV RNA in different body parts of individuals was determined using quantitative RT-PCR, as previously described [25, 29]. Briefly, individual mosquitoes were anesthetized with CO_2_ 14 days after feeding on DENV-containing blood, and the head, wings, and legs separated from the thorax and abdomen under a microscope. Total RNA was extracted from samples using NucleoSpin RNA (Takara Bio, Shiga, Japan). TaqMan Fast Virus 1-Step Master Mix for qPCR (Thermo Fishier Scientific, Waltham, MA USA) was then used to measure the copy number of DENV RNA using the QuantStudio 1 real-time PCR system (Thermo Fishier Scientific). Standard RNAs for each DENV serotype used in this experiment were synthesized in the same manner as previously described [25, 29]. The primer sets and probes used for quantitative RT-PCR, as well as primers used for standard RNA synthesis, are listed in Additional file 1.

In this study, the DENV infection rate (IR) and dissemination rate (DR) were calculated using the following formulae:

$${\text{IR}} = {\text{Number}}\,{\text{of}}\,{\text{individuals}}\,{\text{with}}\,{\text{DENVRN}}\,{\text{A}}\,{\text{detected}}\,{\text{in}}\,{\text{the}}\,{\text{thorax}}\,{\text{and}}\,{\text{abdomen}}/{\text{total}}\,{\text{number}}\,{\text{of}}\,{\text{individuals}}\,{\text{tested}} \times 100.$$

$${\text{DR}} = {\text{Number}}\,{\text{of}}\,{\text{individuals}}\,{\text{with}}\,{\text{DENVRN}}\,{\text{A}}\,{\text{detected}}\,{\text{in}}\,{\text{the}}\,{\text{head}},{\text{wings}},\,{\text{and}}\,{\text{legs}}/{\text{total}}\,{\text{number}}\,{\text{of}}\,{\text{individuals}}\,{\text{tested}} \times 100.$$

### Statistical analyses

Data from the experiments were analyzed using R and GraphPad Prism software (GraphPad Software Inc., San Diego, CA, USA), as well as the Statistics calculators (http://www.statskingdom.com).

## Results

### Artificial blood-feeding and dengue virus infection status in each mosquito colony

DENV propagated in Vero cells resulted in titers of 0.72–7.17 × 10^6^ focus forming units (FFU)/mL in the blood fed on by mosquitoes (Table 3). In the infection experiments, 40 to 50 mosquitoes were obtained in each experimental group 14 days after blood-feeding (Table 4).

**Table 4:** Summary of dengue virus (DENV) infection and dissemination status in mosquito colonies

Species	Colony	DENV serotype	No. of individuals tested	Infection status			Dissemination status		
				No. of infected^a^	IR^b^ (95%CI^c^)	Mean of DENV RNA copies in thorax and abdomen^d^	No. of disseminated^e^	DR^f^ (95% CI)	Mean of DENV RNA copies in head, wings, and legs^d^
*Aedes aegypti*	HCM	1	50	48	96.0 (90.6–100)	8.024	43	86.0 (76.4–95.6)	8.468
		2	50	33	66.0 (46.2–85.8)	7.928	33	66.0 (46.2–85.8)	7.392
		3	50	36	72.0 (46.9–97.1)	7.262	28	56.0 (28.2–83.8)	6.783
		4	50	47	94.0 (77.3–100)	8.108	47	94.0 (77.3–100)	7.458
*Aedes albopictus*	IKT	1	50	15	30.0 (17.3–42.7)	7.817	10	20.0 (8.9–31.1)	7.389
		2	50	10	20.0 (8.9–31.1)	7.298	5	10.0 (0–22.6)	6.949
		3	50	3	6.0 (0–12.6)	6.567	2	4.0 (0–15.0)	7.000
		4	40	15	37.5 (22.5–52.5)	8.154	13	32.5 (0–69.2)	7.032
	BSD	1	50	35	70.0 (57.3–82.7)	8.349	35	70.0 (57.3–82.7)	7.678
		2	48	6	12.5 (3.1–21.9)	7.948	3	6.3 (0–16.6)	5.524
		3	50	13	26.0 (13.8–38.2)	5.611	9	18.0 (0–39.5)	6.572
		4	50	33	66.0 (52.9–79.1)	8.189	33	66.0 (32.8–99.2)	7.433

^a^No. of individuals with DENV RNA detected in the thorax and abdomen

^b^Infection rate (no. of individuals with DENV RNA detected in the thorax and abdomen/ total no. of individuals tested × 100)

^c^95% confidence interval

^d^Mean copy no. expressed in log10

^e^No. of individuals with DENV RNA detected in the head, wings, and legs

^f^Dissemination rate (no. of individuals with DENV RNA detected in the head, wings, and legs/ total No. of individuals tested × 100)

**Fig. 1:**
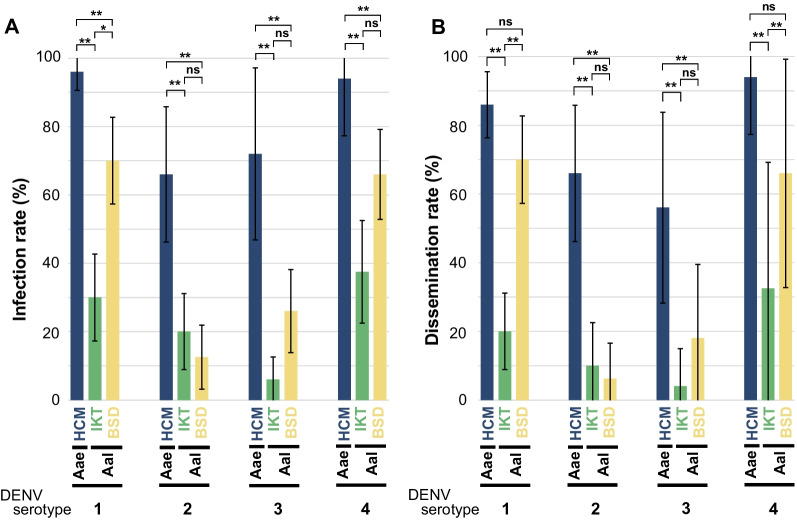
Comparison of the infection and dissemination rates of dengue viruses between mosquito species and colonies. Bars indicate the infection (**A**) and dissemination rates (**B**) at 14 days post-infection with dengue virus (DENV) serotypes in the *Aedes aegypti* (Aae) HCM and *Ae. albopictus* (Aal) IKT and BSD colonies. Error bars represent 95% confidence intervals. Statistical analyses were performed using Fisher's exact test with Bonferroni correction. **, *P* < 0.0001; *, *P* < 0.001; ns, no significant differences (*P* > 0.01)

The IR of all DENV serotypes was significantly higher in the *Ae. aegypti* HCM colony than in both *Ae. albopictus* colonies (Fig. 1A, Table 4). There were differences in the IR between *Ae. albopictus* colonies, with the DENV-1 IR being significantly higher in the BSD colony than in the IKT colony (Fig. 1A, Table 4). The greatest difference in the IR was observed for DENV-3, where the *Ae. aegypti* HCM colony had a 12-fold higher IR than that of the *Ae. albopictus* IKT colony (Table 4).

**Fig. 2:**
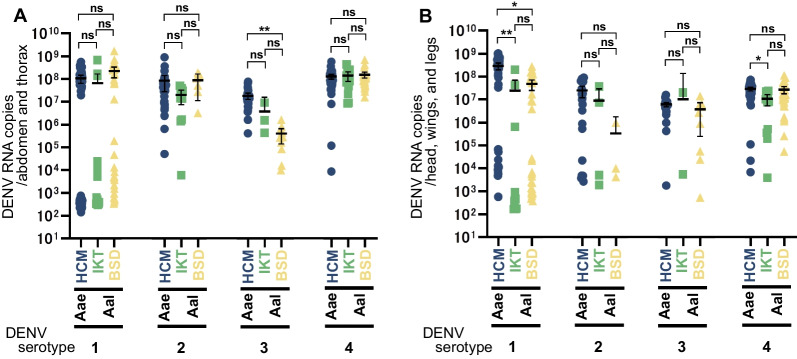
Comparison of dengue virus RNA copy numbers in *Aedes aegypti* and Japanese *Ae. albopictus* colonies. Plots showing the copy numbers of dengue virus (DENV) RNA in the thorax and abdomen (**A)** and the head, wings, and legs (**B)** of individual mosquitoes of the *Aedes aegypti* (Aae) HCM and *Ae. albopictus* (Aal) IKT and BSD colonies 14 days after infection with each DENV serotype. Bars represent the mean with a 95% confidence interval. Statistical analyses were performed using the Mann–Whitney *U* test with Bonferroni correction. **, *P* < 0.0001; *, *P* < 0.001; ns, no significant differences (*P* > 0.01)

Although significant differences in IR were observed between species and colonies, only the *Ae. aegypti* HCM colony had a significantly higher DENV-3 RNA copy number than that of the *Ae. albopictus* BSD colony in the thorax and abdomen; otherwise, there were no significant differences in the DENV RNA copy number between species or colonies (Fig. 2A).

IRs between different serotypes in the same mosquito colony were also compared (Additional file 2). The IR appeared to be influenced by differences in viral titers in the bloodmeal used for the infection experiment (Table 2), but values tended to vary widely between mosquito colonies. Among the DENV serotypes, the highest IRs were observed for DENV-1 in *Ae. aegypti* HCM and *Ae. albopictus* BSD colonies and for DENV-4 in the *Ae. albopictus* IKT colony (Table 4, Additional file 2). The IRs of DENV-1 and DENV-4 were significantly higher than those of DENV-2 and DENV-3 in all colonies (Table 4, Additional file 2). The lowest IR was observed for DENV-2 in the *Ae. aegypti* HCM and *Ae. albopictus* BSD colonies, and for DENV-3 in the *Ae. albopictus* IKT colony (Table 4, Additional file 2). The *Ae. aegypti* HCM colony had IRs > 60% for all serotypes (Table 4, Additional file 2). The *Ae. albopictus* colonies, however, tended to have large differences in IR between serotypes in both colonies, with the greatest difference in observed between serotypes: an approximately 5- to 6-fold difference in IR between DENV-4 and DENV-3 in the IKT colony and between DENV-1 and DENV-2 in the BSD colony (Table 4, Additional file 2).

Furthermore, comparison of viral RNA copy number between different serotypes in the same colony showed that in the thorax and abdomen, the RNA copy number of DENV-4 was significantly higher than that of the other serotypes in the *Ae. aegypti* HCM colony (Additional file 3). Regarding DENV RNA copy number in the thorax and abdomen of the *Ae. albopictus* colonies, DENV-4 was significantly higher than DENV-1 in the IKT colony and DENV-4 was significantly higher than DENV-3 in the BSD colony (Additional file 3). No significant copy number differences were observed between the other serotypes in both *Ae. albopictus* colonies.

### Status of dengue virus dissemination in mosquito species and colonies

In contrast to the IR, no significant differences in dissemination status for DENV-1 and DENV-4 were observed between the *Ae. aegypti* HCM and *Ae. albopictus* BSD colonies; however, both had a significantly higher DR than that of the *Ae. albopictus* IKT colony (Fig. 1B, Table 4). In contrast, for DENV-2 and DENV-3, the *Ae. aegypti* HCM colony had a significantly higher DR than that of both *Ae. albopictus* colonies, similar to the IR results, and no significant differences were observed between the *Ae. albopictus* colonies (Fig. 1B, Table 4). The greatest difference in DR (14-fold) was observed for DENV-3 between *Ae. aegypti* HCM and *Ae. albopictus* IKT colonies (Table 4).

For DENV-1 and DENV-4, DENV RNA was detected in the head, wings, and legs of all individuals in the BSD colony in which viral RNA was detected in the thorax and abdomen (Additional file 4). Similarly, in the *Ae. aegypti* HCM colony, viral RNA was detected in the head, wings, and legs of 100% of the individuals that were positive for DENV-4 RNA in the thorax and abdomen (Additional file 4).

A comparison of DENV RNA copy numbers in the head, wings, and legs between species and colonies revealed that only the DENV-1 copy number was significantly higher in the *Ae. aegypti* HCM colony than in both *Ae. albopictus* colonies (Fig. 2B). The RNA copy number of DENV-4 was significantly higher in the *Ae. aegypti* HCM colony than in the *Ae. albopictus* IKT colony, but otherwise there were no significant differences observed between the species and/or colonies (Fig. 2B).

In addition, DR by serotype was also compared in each colony (Additional file 5). The *Ae. aegypti* HCM colony showed a DR higher than 50% for all serotypes, although there were significant differences among them (Table 4, Additional file 5). Even among the serotypes with the greatest differences in DR, these differences were less than twofold. In the *Ae. albopictus* colonies, however, the difference in DR between serotypes was greater than that observed for IR, with an approximate eightfold difference in DR between DENV-3 and DENV-4 in the IKT colony and an approximate 11-fold difference in DR between DENV-1 and DENV-2 in the BSD colony (Table 4, Additional file 5).

Furthermore, there were almost no significant differences in viral RNA copy numbers in the head, wings, and legs between serotypes, and those of DENV-1 and DENV-4 were significantly higher than that of DENV-3 only in the *Ae. aegypti* HCM colony (Additional file 6).

## Discussion

In this study, all DENV serotypes could infect Japanese *Ae. albopictus* mosquitoes, and their susceptibility to the virus was compared with that of *Ae. aegypti*, the main vector of DENV. The titers of DENV used for infection experiments were 0.72–7.17 × 10^6^ FFU/mL. This is within the range of serum viral titers of imported cases observed in Japan [1.0 × 10^2^–2.9 × 10^7^ plaque forming units (PFU)/mL] [30] and close to the mean viral titer of imported cases (1.3 × 10^7^ PFU /mL) reported in another study [31]. Therefore, the DENV titers used in this infection experiment were considered adequate.

Results of the infection experiments showed that Japanese *Ae. albopictus* was infectious with all DENV serotypes, and viruses were also detected in the head, wings, and legs, indicating that all serotypes exhibited systemic infection. However, the IR of the Japanese *Ae. albopictus*, i.e., viral infection of the thorax and abdomen, including the midgut, was significantly lower than that of *Ae. aegypti* for all DENV serotypes. This suggests that viral infection is inhibited by the midgut infection barrier, which is the first barrier against viral infection [32]. More than half of *Ae. albopictus* individuals with confirmed DENV infections in the thorax and abdomen had DENV RNA detected in their head, wings, and legs, indicating a similar level of dissemination dynamic as that in *Ae. aegypti*. Furthermore, DENV-1 was also found to be more efficiently disseminated in a certain *Ae. albopictus* colony than in *Ae. aegypti*. In addition, there was almost no difference in the number of DENV RNA copies between *Ae. aegypti* and *Ae. albopictus* colonies. This suggests that Japanese *Ae. albopictus* might transmit the virus to the same extent as *Ae. aegypti*, depending on the DENV serotype. However, the extent to which these viruses are expelled with mosquito saliva was not investigated in this study; therefore, further research is needed to confirm the ability of Japanese *Ae. albopictus* to transmit the DENV serotypes.

This study showed that Japanese *Ae. albopictus* have large differences in IR and DR among the DENV serotypes. This is consistent with data observed in previous studies on *Ae. albopictus* that show differences in susceptibility to DENV serotypes [33, 34]. The results of the present study confirmed that DENV-1 and DENV-4 infected both *Ae. albopictus* colonies more efficiently than serotypes 2 and 3. However, the virus titers used in the infection experiments in this study differed between serotypes, and it is possible that differences in the initial amount of virus sucked by the mosquitoes may have affected their subsequent susceptibility. Moreover, in this study, only a certain of the many genotypes of each DENV serotype were used in the experiments. Previous studies have reported that mosquito susceptibility to different viral genotypes within the same serotype also varies [35–37]. Therefore, we expect that future studies using genotypes other than those used in this study could reveal more detailed differences in the susceptibility of Japanese *Ae. albopictus* to different DENV serotypes.

To date, several outbreaks of dengue fever have been reported in temperate zones in Japan and Europe, where *Ae. albopictus* was the only mosquito vector (Table 1). Despite the identification of imported cases with different DENV serotypes in these regions [38, 39], the majority of autochthonous epidemics have been caused by DENV-1 (Table 1). Additionally, DENV-1 is the only or major epidemic serotype caused in dengue epidemics even in tropical and subtropical regions where *Ae. albopictus* was the sole vector mosquito [8–10]. Thus, many outbreaks of *Ae. albopictus* as the main vector were caused by DENV-1. Since DENV-1 used in this study is a Japanese epidemic strain [28], the possibility that it was already adapted to *Ae. albopictus* cannot be ruled out, but it showed high infectivity and propagation in Japanese *Ae. albopictus* among the serotypes tested. This suggests that *Ae. albopictus* is highly compatible with DENV-1. Therefore, DENV-1 is more likely to spread during an epidemic in which *Ae. albopictus* is the primary vector. In addition, results of this study indicated that *Ae. albopictus* is as highly susceptible to DENV-4 as it is to DENV-1. To date, DENV-4 has not been prevalent in outbreaks in which *Ae. albopictus* was the primary vector. However, based on results of the present study, there may be a risk of future outbreaks of this serotype in areas where *Ae. albopictus* is the dominant vector.

## Conclusions

In the present study, we investigated the susceptibility of Japanese *Ae. albopictus* to DENV and compared its IR, DR, and DENV propagation efficiency with those of *Ae. aegypti*, the main vector of DENV. The analyses revealed for the first time that Japanese *Ae. albopictus* was susceptible to all DENV serotypes. Compared with that of *Ae. aegypti*, a higher percentage of Japanese *Ae. albopictus* had an inhibitory effect on DENV infection via the midgut infection barrier. However, once the virus overcomes this barrier, it propagates and disseminates to the hemocoel and other tissues in *Ae. albopictus* as efficiently as that in *Ae. aegypti*. Based on previous dengue outbreak trends and the results of the infection experiment in this study, *Ae. albopictus* is predicted to be highly compatible with DENV-1, suggesting that this serotype poses a high risk for future epidemics in Japan.

### Supplementary Information

**Additional file 1.** List of primers and probes used in this study.

**Additional file 2.** Comparison of the infection rates of dengue virus serotypes in each mosquito species and colony.

**Additional file 3.** Comparison of dengue virus serotype propagation in *Aedes aegypti *and Japanese *Ae. albopictus *colonies.

**Additional file 4.** Dissemination rate of *Aedes aegypti *and Japanese *Ae. albopictus *colonies.

**Additional file 5.** Comparison of the dissemination rates of dengue virus serotypes in each mosquito species and colony.

**Additional file 6.** Comparison of dengue virus serotype propagation in *Aedes aegypti *and Japanese *Ae. albopictus *colonies.

## Data Availability

The datasets supporting the conclusions of this article are included within the article and its additional files.

## Abbreviations

D: Dark
DENV: Dengue virus
DENV-1: Dengue virus serotype 1
DENV-2: Dengue virus serotype 2
DENV-3: Dengue virus serotype 3
DENV-4: Dengue virus serotype 4
DR: Dissemination rate
FFU: Focus forming units
IR: Infection rate
L: Light
PFU: Plaque forming units
RT-PCR: Reverse transcription polymerase chain reaction
