# Supplementary material for: Comparative analysis of the susceptibility of Aedes aegypti and Japanese Aedes albopictus to all dengue virus serotypes
Source: Trop Med Health. 2023 Nov 2;51:61. doi: 10.1186/s41182-023-00553-5 (PMC10621184; doi:10.1186/s41182-023-00553-5)
Supplement: Supplementary file 1 — Additional file 1. List of primers and probes used in this study. [file 41182_2023_553_MOESM1_ESM.docx]

Additional file 1: List of primers and probes used in this study.

| Serotype | Experiment | Primer or probe name | Sequence (5'-3') |
| --- | --- | --- | --- |
| 1 | quantitative RT-PCR | D1MGBEn469s | GAACATGGRACAAYTGCAACYAT |
|  |  | D1MGBEn536r | YCCGTAGTCDGTCAGCTGTATTTC |
|  |  | D1MGBEn493p (probe) | ACACCTCAAGCTCC |
|  | strandard RNA synthesis | D1Es-FW | GTGCTAAGTTCAAGTGTGTGACA |
|  |  | D1EsT7-FW | TAATACGACTCACTATAGGGGTGCTAAGTTCAAGTGTGTGACA |
|  |  | D1Es-RV | CAAGGCAGTGGTAGGTCTAGAAA |
| 2 | quantitative RT-PCR | D2MGBEn493s | ACACCACAGAGTTCCATCACAGA |
|  |  | D2MGBEn568r | CATCTCATTGAAGTCNAGGCC |
|  |  | D2MGBEn545p (probe) | CGATGGARTGCTCTC |
|  | strandard RNA synthesis | DENV2staF | CCATTGTGATAACACCTCACTCA |
|  |  | DENV2T7staF | TAATACGACTCACTATAGGGCCATTGTGATAACACCTCACTCA |
|  |  | DENV2staR | ATCCAATTTGATCCTTGTGTGTC |
| 3 | quantitative RT-PCR | DEN-3(4P)s | GGACTGGACACACGCACTCA |
|  |  | DEN-3(4P)r | CATGTCTCTACCTTCTCGACTTGTCT |
|  |  | DEN3p-Barbara (probe) | ACCTGGATGTCGGCTGAAGGAGCTTG |
|  | strandard RNA synthesis | DENV3staF | AAGTGGAACCTGAAGACATTGAC |
|  |  | DENV3T7staF | TAATACGACTCACTATAGGGAAGTGGAACCTGAAGACATTGAC |
|  |  | DENV3staR | AGGTGCCTATGTAATGAGCAAGA |
| 4 | quantitative RT-PCR | D4TEn711s | GGTGACRTTYAARGTHCCTCAT |
|  |  | D4TEn786c | WGARTGCATRGCTCCYTCCTG |
|  |  | D4TEn734p (probe) | CCAAGAGACAGGATGTGACAGTGCTRGGATC |
|  | strandard RNA synthesis | DENV4staF | GCAGACACATCAGAAGTTCATTG |
|  |  | DENV4T7staF | TAATACGACTCACTATAGGGGCAGACACATCAGAAGTTCATTG |
|  |  | DENV4staR | ATGTGATTTCCATCACCAGAATC |
