# Supplementary material for: Comparative analysis of the susceptibility of Aedes aegypti and Japanese Aedes albopictus to all dengue virus serotypes
Source: Trop Med Health. 2023 Nov 2;51:61. doi: 10.1186/s41182-023-00553-5 (PMC10621184; doi:10.1186/s41182-023-00553-5)
Supplement: Supplementary file 2 — Additional file 2. Comparison of the infection rates of dengue virus serotypes in each mosquito species and colony. [file 41182_2023_553_MOESM2_ESM.pdf]

Additional file 2

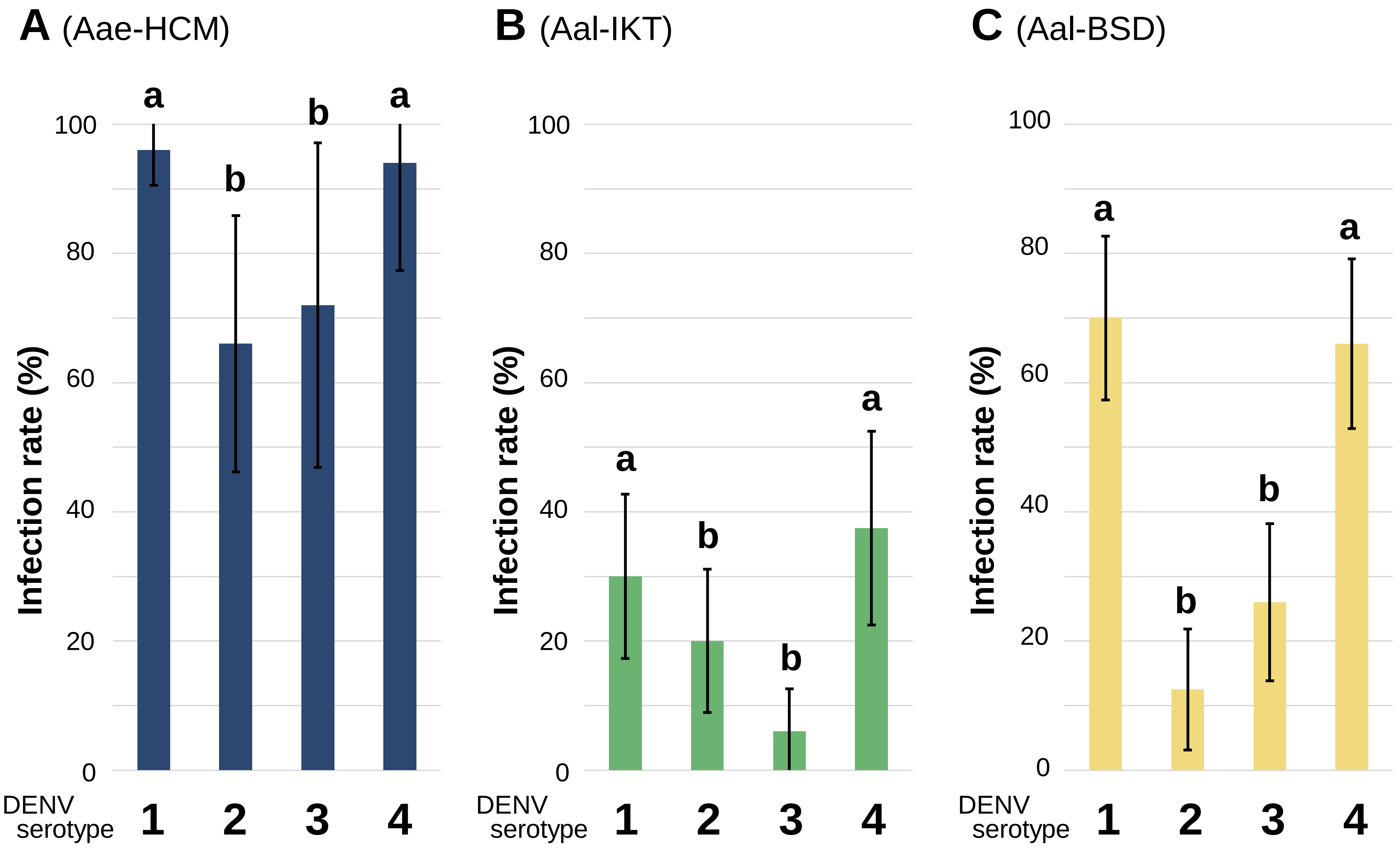

Additional file 2 legend

Comparison of the infection rates of dengue virus serotypes in each mosquito species and colony.
The infection rate of dengue virus (DENV) serotypes for each mosquito species and colony is shown in bar graphs, which was determined by detecting DENV RNA from the thorax and abdomen of individual mosquitoes 14 days after feeding on blood containing the virus. A, B, and C show results for the *Aedes aegypti* (Aae) HCM and *Ae. albopictus* (Aal) IKT and BSD colonies, respectively. Error bars represent 95% confidence intervals. Statistical analyses were performed using Fisher's exact test with Holm-Bonferroni correction. Statistically significant differences ($P < 0.01$) between DENV serotypes are indicated by letters (a or b). Serotypes with the same letter in each graph are not significantly different.
