# Supplementary material for: Comparative analysis of the susceptibility of Aedes aegypti and Japanese Aedes albopictus to all dengue virus serotypes
Source: Trop Med Health. 2023 Nov 2;51:61. doi: 10.1186/s41182-023-00553-5 (PMC10621184; doi:10.1186/s41182-023-00553-5)
Supplement: Supplementary file 3 — Additional file 3. Comparison of dengue virus serotype propagation in Aedes aegypti and Japanese Ae. albopictus colonies. [file 41182_2023_553_MOESM3_ESM.pdf]

Additional file 3

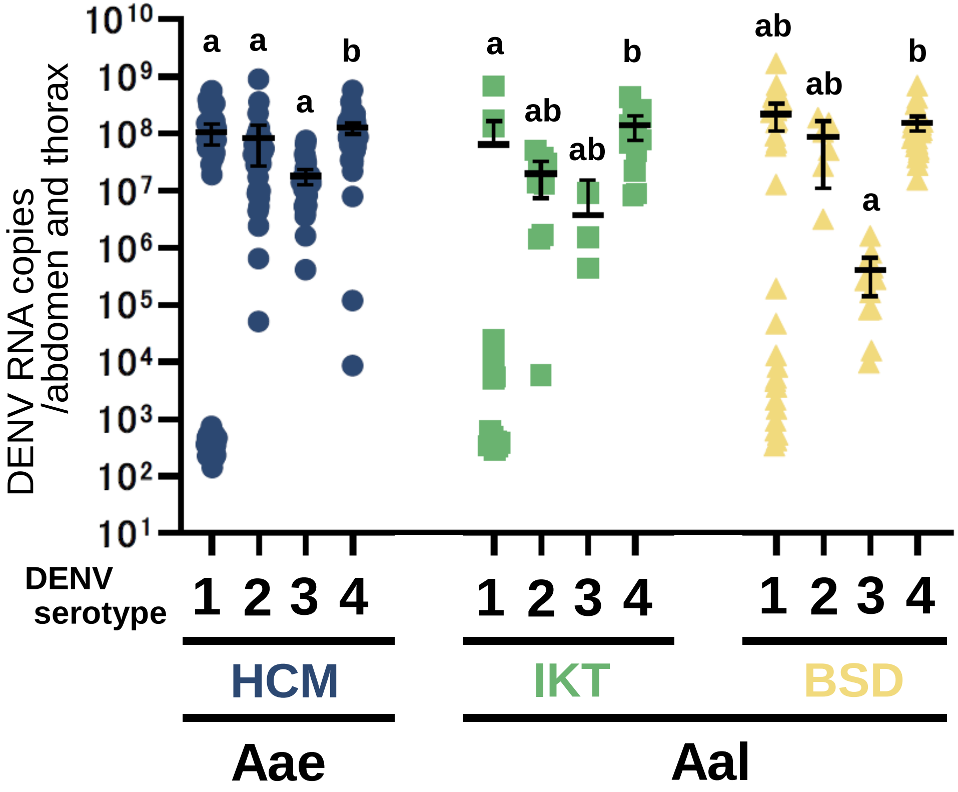

Additional file 3 legend

Comparison of dengue virus serotype propagation in *Aedes aegypti* and Japanese *Ae. albopictus* colonies.

Plots show copy numbers of dengue virus (DENV) RNA in the thorax and abdomen of individual mosquitoes of the *Aedes aegypti* (Aea) and *Ae. albopictus* (Aal) colonies 14 days after infection with each DENV serotype. Error bars indicate 95% confidence intervals for the mean viral RNA copy number. Statistical analyses were performed using the Mann-Whitney U test with Holm-Bonferroni correction. Statistically significant differences (P <0.01) between DENV serotypes are indicated by letters (a, b, ab, ac, or bc). Serotypes with the same letter in each graph are not significantly different.
