# Supplementary material for: Comparative analysis of the susceptibility of Aedes aegypti and Japanese Aedes albopictus to all dengue virus serotypes
Source: Trop Med Health. 2023 Nov 2;51:61. doi: 10.1186/s41182-023-00553-5 (PMC10621184; doi:10.1186/s41182-023-00553-5)
Supplement: Supplementary file 4 — Additional file 4. Dissemination rate of Aedes aegypti and Japanese Ae. albopictus colonies. [file 41182_2023_553_MOESM4_ESM.pdf]

# Additional file 4

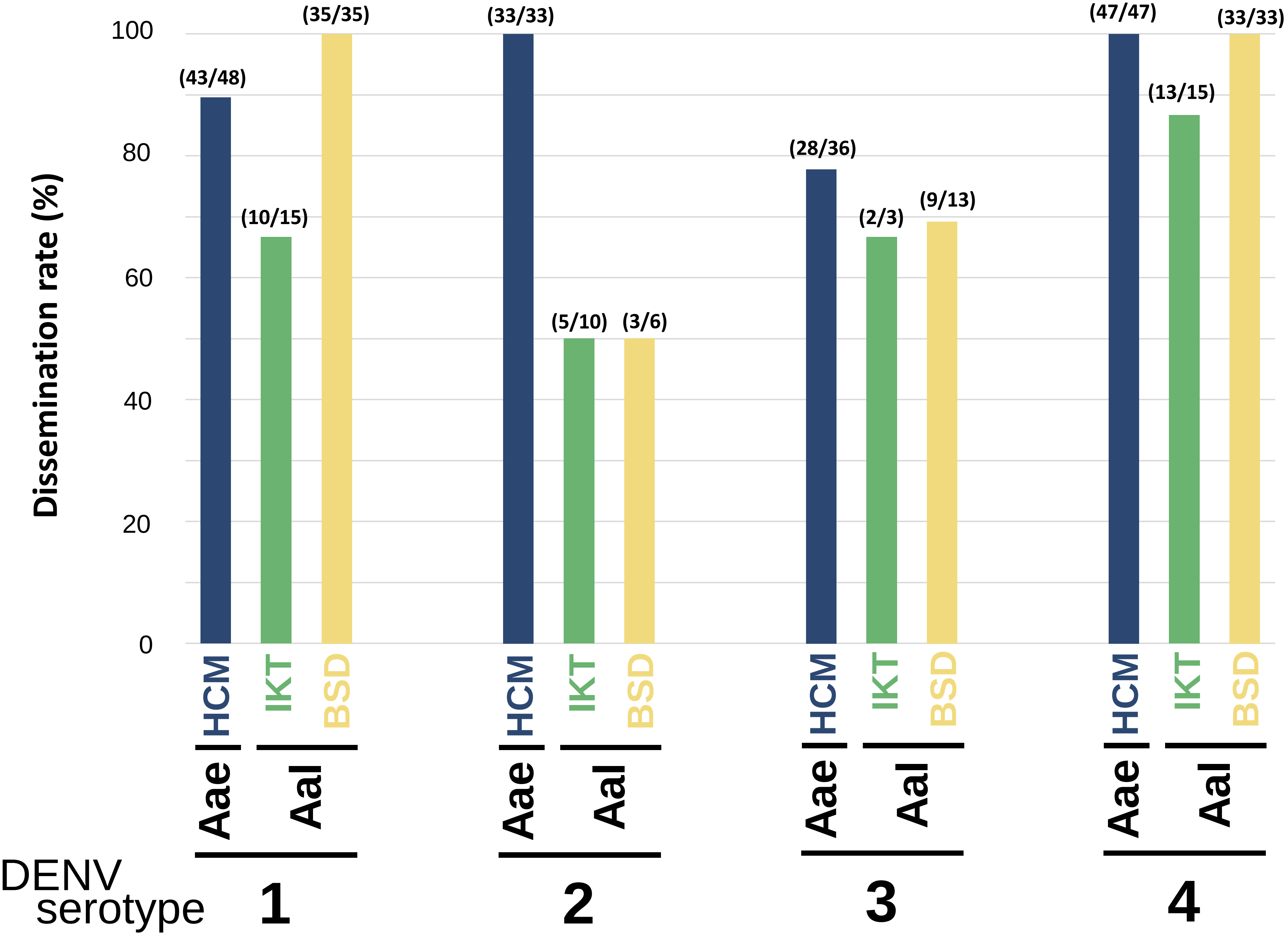

## Additional file 4 legend

**Dissemination rate of *Aedes aegypti* and Japanese *Ae. albopictus* colonies.**

The dissemination rate for each mosquito species and colony, based on the population of individual mosquitoes in which dengue virus (DENV) RNA was detected in the thorax and abdomen, is shown in the bar graph. Numbers in parentheses at the top of each bar indicate the following: (number of individuals with DENV RNA detected in the head, wings, and legs/number of individuals with DENV RNA detected in the thorax and abdomen).
